# Alterations of m6A RNA methylation regulators contribute to autophagy and immune infiltration in primary Sjögren’s syndrome

**DOI:** 10.3389/fimmu.2022.949206

**Published:** 2022-09-20

**Authors:** Linlin Cheng, Haolong Li, Haoting Zhan, Yongmei Liu, Xiaomeng Li, Yuan Huang, Li Wang, Fengchun Zhang, Yongzhe Li

**Affiliations:** ^1^ Department of Clinical Laboratory, State Key Laboratory of Complex Severe and Rare Diseases, Peking Union Medical College Hospital, Chinese Academy of Medical Science and Peking Union Medical College, Beijing, China; ^2^ Department of Medical Research Center, Peking Union Medical College Hospital, Chinese Academy of Medical Science and Peking Union Medical College, Beijing, China

**Keywords:** primary Sjögren’s syndrome, m6A, autophagy, immune microenvironment, GEO

## Abstract

N6-methyladenosine (m6A) RNA modification is a new epigenetic regulation mechanism on eukaryotic mRNA. Few autoimmune diseases focused on the role of m6A in their pathogenies, and m6A modulation in the pathological process of primary Sjögren’s syndrome (pSS) is still unknown. In this work, three microarray datasets of pSS patients were downloaded from the GEO database: datasets #1 and #2 from the whole peripheral blood (PB) samples, dataset #3 from the labial salivary gland tissue samples, as well as a PB cohort collected from our hospital. Six differentially expressed m6A regulators were identified by comparing the PB dataset #1 of pSS and healthy controls using the Wilcox test and logistic regression analysis. Among them, four (ALKBH5, RBMX, RBM15B, and YTHDF1) were confirmed as down-regulated in PB dataset #2 and in our PB cohort by RT-PCR, and four (ALKBH5, METTL3, RBM15B, and YTHDF1) were confirmed as down-regulated in the dataset #3 of the labial gland tissue. In addition, discrepantly expressed m6A regulators accompanied by diverse immunocytes, including dendritic cells (DCs), T cells, and CD56dim natural killer cells, and among the regulators, ALKBH5 and METTL3 were comprehensively linked with the infiltrated immune cells. Notably, the most enriched autophagy mechanism mediated by m6A was observed in pSS using functional annotation analysis. Ten hub genes were identified using a protein-protein interaction network, and their expression in PB dataset #2 and the expression of three genes (PIK3CA, STAT1, and MAPK3) in the labial gland tissue dataset #3 were confirmed. Our study provides evidence that m6A methylation is widely involved in the immune infiltration and autophagy of pSS, thus contributing to the pathogenesis of this disease and potentially representing a novel therapeutic target.

## Introduction

Sjögren’s syndrome (SS) is a systemic autoimmune disease that mainly affects the exocrine glands, and it is characterized by dryness of the eyes and mouth (1). Genetic and environmental factors may contribute to its occurrence and development (2). Activated epithelial cells of the salivary gland represent the initiating factor that leads to the immune and inflammatory disorders in pSS, immunocytes infiltration, as well as amplified production of autoantibodies and interferon alpha, consequently leading to tissue damage (2). Thus, pSS is also defined as “autoimmune epithelitis” (3). Nonetheless, the pathological mechanisms regulating pSS remain unclear.

N6-methyladenosine (m6A) RNA modification has emerged in recent years as a new epigenetic regulatory mechanism influencing mRNA stability, translation, translocation, and control of gene expression in eukaryotes. It is a reversible process driven by three groups of enzymes called “writers” (methyltransferases including METTL3, METTL14, METTL16, and WTAP), “erasers” (demethylases including FTO and ALKBH5), and “readers” (m6A-binding proteins, including YTHDC1, YTHDC2, YTHDF1, YTHDF2, FMR1, and RBMX) (4). m6A plays important roles in almost all vital biological processes, including the inflammatory and antitumor immune response, antiviral immunity, and T-cell homeostasis, therefore affecting both health and diseases (5, 6). Some studies demonstrated the key role of m6A regulators in tumor initiation, progression, and metastasis by disturbing the balance between apoptosis, differentiation, and proliferation (7), or by regulating glycolytic metabolism to evade immune surveillance (8), or controlling the interaction between cancer cells and microenvironment to promote metastasis (9).

In recent years, m6A modification has attracted more and more attention in a wide range of non-cancer areas. Nie et al. found that m6A regulators interact with risk genes of inflammatory bowel diseases (10). Zhang et al. reported that m6A modification plays a crucial role in the abundance of monocytes and multiple immune reactions involving TNF family member receptors and cytokines in patients with periodontitis (11). Li et al. demonstrated that m6A regulators are related to the degree of immune infiltration of central memory T cells, macrophages, mast cells, gamma delta T cells, and NK CD56 bright cells in patients with abdominal aortic aneurysms. To be specific, m6A-modified genes are involved in body metabolism and autophagy (12). All this weight of evidence implies that aberrant m6A modification may underlie the autoimmunity and dysfunction of other important pathological processes through various mechanisms (13, 14). However, few autoimmune diseases focused on the role of m6A in their pathogenesis (15–20), and the mechanism of action of m6A in the pathological process of pSS is still unknown.

Nowadays, the public database has become a promising resource for extracting valuable data. Therefore, this study evaluated several datasets from the GEO database and our collected cohort covering the peripheral blood (PB) and salivary gland samples to analyze the expression pattern of m6A regulators in pSS. The aim of this study was to uncover the key m6A regulators by bioinformatic tools, acting as diagnostic biomarkers and their involvement in vital biological mechanisms for an epigenetic-based therapy in pSS.

## Methods

### Data collection

Three datasets (dataset #1: GSE51092; dataset #2: GSE84844; dataset #3: GSE40568) were downloaded from gene expression omnibus (GEO) using the search words “((Sjögren’s syndrome) OR (Sjogren syndrome)) AND microarray expression data AND Homo sapiens”. Dataset #1 was the discovery set composed of 222 whole PB samples from 190 pSS and 32 healthy controls (HCs) samples; the sequencing for the expression profile was performed on the Human WG-6 BeadChip microarray (Illumina, San Diego). Dataset #2 was composed of 60 PB samples from 30 pSS and 30 HCs; the sequencing for the expression profile was performed on the Affymetrix Human Genome U133 plus 2.0 Array (Affymetrix, Santa Clara, California, USA). Dataset #3 was composed of 8 samples from labial salivary glands biopsy of 5 pSS and 3 HCs; the sequencing for the expression profile was performed on the Affymetrix GeneChip Human Genome U133 plus 2.0 Array (Affymetrix, Santa Clara, California, USA). The gene matrix of each dataset was normalized by the “normalizeBetweenArrays” function in the “limma” package. The gene list of m6A regulators was obtained from previous studies (4, 11, 21).

### m6A signature in pSS

Protein-protein interaction (PPI) network of 26 m6A regulators was constructed using STRING (https://cn.string-db.org/). The correlation matrix analysis was performed to visualize the association between m6A regulators, and rectangles of clustered m6A regulators on the matrix were drawn based on the hierarchical cluster (hclust). The correlation analysis of m6A “writers” and “erasers” with the significant spearman correlation r > 0.3 (*p* < 0.05) was presented.

### Differential expression of m6A regulators

The expression of m6A regulators between pSS and HCs samples from dataset #1, and between groups of pSS patients with high and low anti-Ro/SSA antibody levels (high: higher than the median level, low: lower than the median level) in dataset #2 was compared using the Wilcox test. A volcano plot was drawn to visualize the different profiles in the expression in m6A regulators (DEMRs). The heatmap was constructed to show the expression of DEMRs in pSS and HCs. The univariate logistic regression was performed to identify the m6A regulators related to pSS, and multivariate logistic regression analysis was performed to construct the classifier for pSS from HCs by DEMRs. The receiver operating characteristic (ROC) curve analysis was used to evaluate the distinguishing performance of the m6A regulator panel. The identified DEMRs from the dataset #1 were validated in the dataset #2 (PB samples), dataset #3 (samples of labial salivary glands biopsy) and our PB cohort.

### Construction of the nomogram

A nomogram was constructed using the “rms” R package to quantify the predicted risk for pSS based on the results of the logistic regression analysis through a simple visualization figure (22). The calibration and decision curve analyses (DCA) were used to determine the performance of the nomogram. The calibration curves were graphically assessed with a bootstrap of 1000 samples by mapping the nomogram-predicted probabilities against the observed rates, and the 45° line represented the best predictive values. The “rmda” package was used for a net benefit-dependent DCA, which is a novel method for assessing clinical predictive models by examining the range of threshold probabilities and the overall therapeutic advantage (23).

### Single-sample gene set enrichment analysis of immune characteristics

Single-sample gene-set enrichment analysis (ssgsea) was performed using the “GSVA” R package to assess the enrichment score of 23 specific immunocytes (24) and immune reactions for each sample within a given data set. The gene-set containing 17 types of immune reactions was downloaded from the ImmPort database (http://www.immport.org). The diverse immunocyte abundance and the immune reaction between groups were identified according to the enrichment scores using the Wilcox test. The correlation matrix between DEMRs and immune scores was constructed by Spearman correlation analysis.

### Functional annotation analysis of genes mediated by DEMRs

The target genes of DEMRs were predicted by searching them in the m6A2Target database (http://m6a2target.canceromics.org/#/). Spearman correlation analysis was performed to search the co-expressed genes of DEMRs using the criteria r > 0.3 and *p* < 0.01. The genes mediated by DEMRs in pSS were defined as the intersection of the predicted target genes and the co-expressed genes. The functional annotation analysis (FAA), including gene ontology (GO) annotation incorporated by biological process (BP), cellular components (CC), and molecular functions (MF), as well as the Kyoto encyclopedia of genes and genomes (KEGG) pathway enrichment analysis were performed using the “clusterProfiler” R package to obtain the biological functions and signaling pathways of genes mediated by DEMRs. The criterion to evaluate the significant terms was set as a q value less than 0.05.

### Identification of the differentially expressed genes between groups

The differentially expressed genes (DEGs) between pSS and HCs samples from dataset #1, and between groups of pSS patients with high and low anti-Ro/SSA antibody levels in dataset #2 were identified using the lmFit and contrasts.fit functions of the limma package. The empirical eBayes command in the limma package was used to calculate the consensus pooled variance of genes and adjust the associated *p*-value. Genes with logFC absolute values ≥ log2 (1.2) and *p*-value <0.05 were defined as DEGs.

### Protein-protein interaction network and hub gene of autophagy

Taken together, 1027 unique genes involved in autophagy were obtained by integrating the gene sets from GO_Autophagy, KEGG_ Autophagy, and HAMdb database (http://hamdb.scbdd.com/). The HAMdb database contains 796 genes including 545 genes from the literature and 251 collected from online databases.

The confidence of 0.15 was set as the minimum required interaction score, and the protein-protein interaction (PPI) network of DEMRs and autophagy-related genes was developed using STRING to unveil the m6A modified autophagy genes. In addition, the PPI network of autophagy-related genes and differentially expressed genes in pSS setting confidence > 0.4 was constructed. PPI maps were then visualized using Cytoscape (Version 3.9.1). CytoHubba plugin was applied to extract the top 10 key nodes/hubs ranked by maximum clique centrality (MCC). Molecular Complex Detection (MCODE) plugin was used to detect densely connected regions in large PPI networks (25) by parameters keeping a degree cut-off = 0.2, node score cut-off = 0.2, k-score = 2, and max.Depth = 100, as previously described (26), and the top 3 MCODE clusters were retrieved. The association between the hub genes of autophagy and DEMRs was analyzed using the spearman correlation analysis and visualized using the chord diagram.

### Validation of DEMRs and autophagy hub genes

Dataset #2 and dataset #3 were used to validate the 6 DEMRs and 10 hub genes of autophagy identified in dataset #1. Their expression between pSS and HC PB/tissue samples was compared using the Wilcox test.

### Patients and samples

pSS predominantly affects women and has peak incidence at 50 years old (1). Therefore, the whole blood samples were obtained from 17 women with pSS and 17 age-matched female HCs at the Peking Union Medical College Hospital (PUMCH) from March 2022 to April 2022. This study was approved by the Medical Ethics Committee of PUMCH and informed consent was obtained from the enrolled subjects (JS-2049). The clinical characteristics of the subjects are listed in **Supplementary Table 1**.

### RNA isolation and RT-PCR

Total RNA was isolated from the whole blood samples of the enrolled subject at the day of blood collection according to the manufacturer’s instructions (Mei5 Biotechnology, Co., Ltd). Then, the RNA samples were preprocessed using gDNA plus remover mix to remove genomic DNA contamination, and then, the RNA was reverse transcribed to cDNA using M5 RT Super plus Mix. HiPerSYBR Premix Estaq (Mei5 Biotechnology, Co., Ltd) was used to perform the real-time PCR on a Roche LightCycler 480 system, and the relative expression of the genes was calculated using the 2^−ΔΔCt^ method after the normalization with the endogenous GAPDH mRNA expression. The primer sequences of the 6 DEMRs are listed in **Table 1**.

**Table 1:** Primer sequences for real-time PCR.

Gene	Forward primer	Reverse primer
METTL3	AAGCTGCACTTCAGACGAAT	GGAATCACCTCCGACACTC
RBM15B	GGGAGCATTCGGACCATTGA	CTCATTTTAGCACAGGCGGC
YTHDC2	GGTATCCCCTGCCGTATATTTTG	CTTTCCCGTCTCTCTGCGG
YTHDF1	ATACCTCACCACCTACGGACA	GTGCTGATAGATGTTGTTCCCC
RBMX	TGGAAGCAGTCGCTATGATG	GAGGGTACCCCCTTTCCATA
ALKBH5	CCCGAGGGCTTCGTCAACA	CGACACCCGAATAGGCTTGA
GAPDH	CCTCAAGATCATCAGCAAT	CCATCCACAGTCTTCTGGGT

### Association of m6A regulators and autophagy hub genes with clinical features

The association of DEMRs and autophagy hub genes with clinical features of pSS was found by Spearman analysis based on dataset #2, including age, gender, rheumatoid factor, antinuclear antibodies (ANA), IgG, IgA, IgM, anti-Sjögren’s-syndrome-related antigen A autoantibodies (anti-SSA), ant-Sjögren’s-syndrome-related antigen B autoantibodies (anti-SSB), and EULAR Sjögren’s syndrome disease activity index (ESSDAI), to investigate the clinical value of these genes in the disease activity, immune response, and diagnostics.

## Results

### Overview of m6A regulator signature in pSS

**Figure 1:**
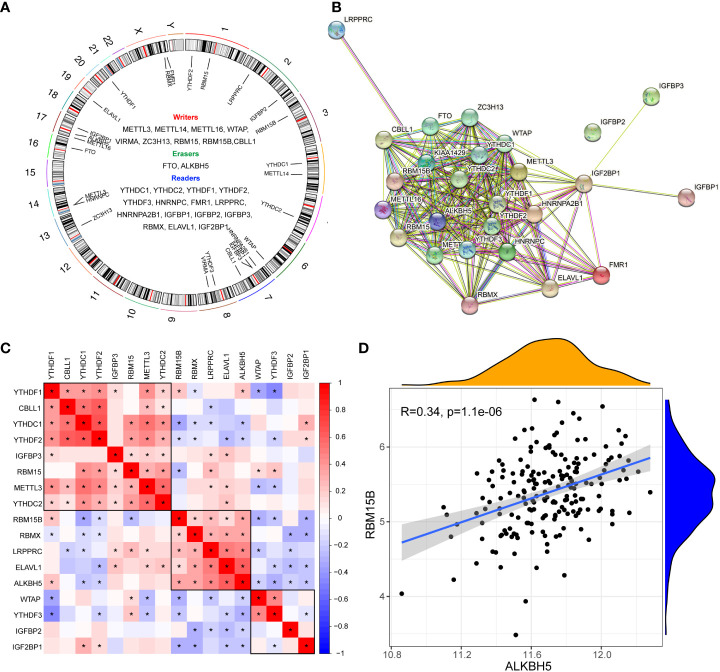
Expression landscape of m6A regulators in primary Sjögren’s syndrome. **(A)** Circus diagram indicating the location of m6A regulators in the chromosome. **(B)** The protein-protein interaction network of m6A regulators. **(C)** The correlations among the expression of 27 m6A regulators in pSS PB samples. **(D)** The scatter plot demonstrated the correlations between dysregulated m6A “writers” and m6A “erasers” with r>0.3 and p<0.01; *, p<0.05.

The gene list of 26 m6A regulators was obtained from previous studies (4, 11, 21) (**Figure 1A**). The PPI network of the m6A regulators demonstrated their compact interactions and synergistic effect (**Figure 1B**). Altogether, 17 m6A regulators were detected in dataset #1, including 5 “writers” (METTL3, WTAP, RBM15, RBM15B, and CBLL1), 1 “eraser” (ALKBH5), and 11 “readers” (YTHDC1, YTHDC2, YTHDF1, YTHDF2, YTHDF3, LRPPRC, IGFBP2, IGFBP3, RBMX, ELAVL1, and IGF2BP1). Furthermore, the correlation matrix of the 17 m6A regulators in pSS revealed the universal correlation and their function as a complex (**Figure 1C**). The “writer” RBM15B showed a close association with the “eraser” ALKBH5” with a coefficient r = 0.34 (*p* < 0.0001) (**Figure 1D**).

### Identification of pSS-related m6A regulators

**Figure 2:**
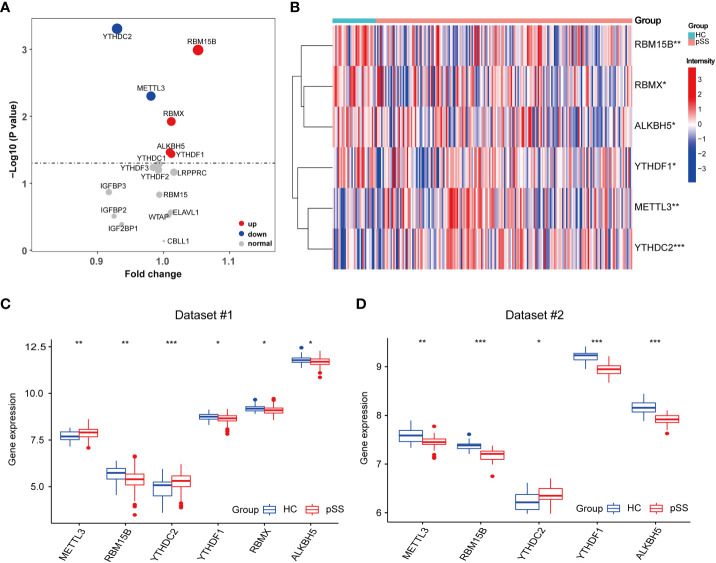
Identification of pSS-related m6A regulators. **(A)** The volcano plot illustrates the m6A regulators that are dysregulated between the HCs and pSS groups. Upregulated and downregulated significant genes (p < 0.05) are indicated in red and blue dots, respectively. B-D. The heatmap **(B)** and the box plots **(C, D)** demonstrated the transcriptome expression status of the 6 DEMRs between healthy and pSS PB samples in dataset #1 **(B, C)** and dataset #2 **(D)**. *, p<0.05; **, p<0.01; ***, p<0.001.

The comparison of 17 m6A regulators from PB samples of pSS and HCs was performed. The volcano plot shows that the expression of 6 m6A regulators was different; 4 were up-regulated (RBM15B, RBMX, ALKBH5, and YTHDF1) and 2 were down-regulated (YTHDC2 and METTL3) in pSS compared to HCs (**Figure 2A**). The expression pattern of the 6 DEMRs is shown in **Figures 2B**, **C**. All DEMRs in validation dataset #2 were validated except RBMX, which was not detected by the Affymetrix Human Genome U133 plus 2.0 Array (**Figure 2D**). However, METTL3 showed a significantly opposite trend compared with that in the dataset #1.

**Figure 3:**
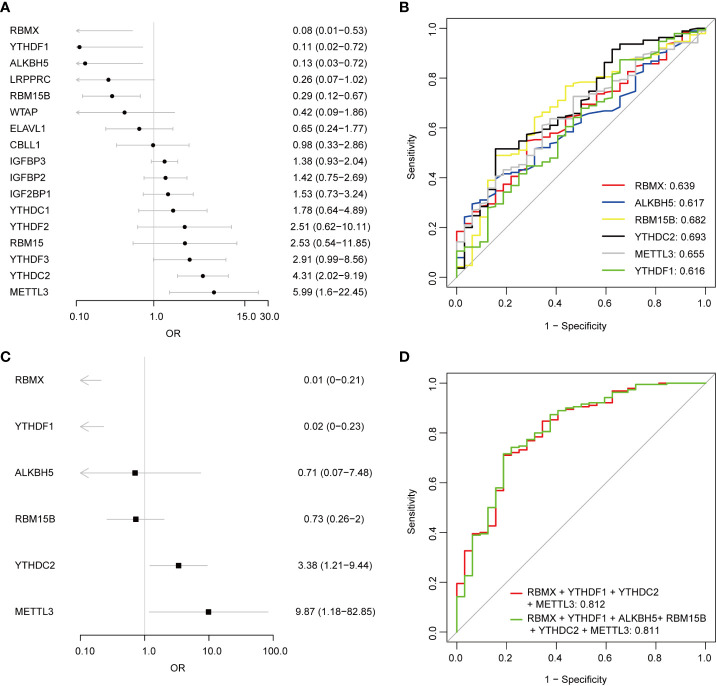
Logistic regression models investigating the relationship between m6A regulators and pSS. **(A)** Univariate logistic regression investigated the relationship between m6A regulators and pSS. **(B)** The discrimination ability of the 6 DEMRs for HCs and pSS samples was analyzed by ROC curve and AUC value. **(C)** Distinguishing signature was developed by multivariate logistic regression. **(D)** The discrimination ability of the m6A regulator panels for HCs and pSS samples was analyzed by ROC curve and AUC value.

Univariate logistic regression analysis was performed to investigate the contribution of the 17 detected m6A regulators to the pathogenesis of pSS (**Figure 3A**). Six m6A regulators were essential for pSS in the univariate logistic regression consistently with the differentially expressed analysis. The ROC analysis indicated that each DEMR discriminated pSS from HC with an AUC > 0.6 (**Figure 3B**). Multivariate logistic regression analysis was performed to develop a quaternary classifier to distinguish pSS and HCs (**Figure 3C**). The ROC curve verified that the quaternary panels composed of METTL3, YTHDC2, YTHDF1, and RMBX presented the best performance in distinguishing pSS from HCs (AUC = 0.812), revealing the involvement of m6A regulators in the pathogenesis of pSS (**Figure 3D**).

### Construction of a nomogram based on DEMRs

A nomogram was constructed using 6 DEMRs to provide a quantitative approach for clinicians to predict the risk of pSS (**Supplementary Figure 1A**). In this nomogram based on the logistic regression model, a scale was used to assign points to the 6 DEMRs. A straight line was drawn upward to determine the points for each DEMR. The points of all the 6 DEMRs were accumulated as the “Total Points”. The probability of pSS was determined by drawing a vertical line from the “Total Points” axis straight downwards to the axis of the “Risk of Disease”. RBMX and YTHDF1 contributed to the most risk points compared with the other DEMRs, and this result was consistent with our logistic regression results. The bias-corrected line in the calibration chart was close to the ideal curve (the 45-degree line), suggesting a good consistency between the observation and prediction (**Supplementary Figure 1B**). The DCA showed that the nomogram had a high clinical application value (**Supplementary Figure 1C**). These findings suggested that the nomogram represented a better tool for predicting pSS in clinically suspected patients.

### m6A regulators were associated with immune dysfunction in pSS

**Figure 4:**
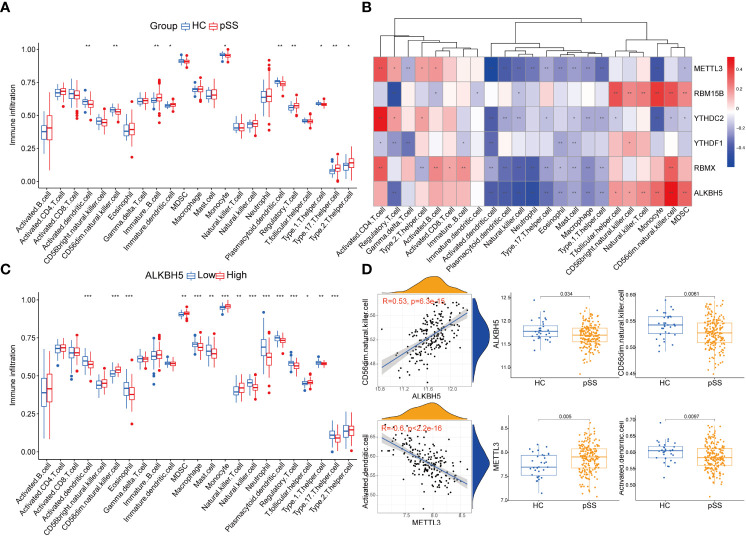
Immune microenvironment characteristics in pSS. **(A)** The immune score differences of each immune microenvironment infiltrating immunocyte in HCs and pSS. **(B)** The correlation matrix between the immune score and the 6 DEMRs. **(C)** The correlation between immune score and ALKBH5. **(D)** ALKBH5 and METTL3 showed a comprehensive linkage with the infiltrating immune cells. As for ALKBH5, the most correlated pair is ALKBH5 - CD56dim natural killer cells, the expression differences of ALKBH5 and activated dendritic cells in HCs and pSS is presented by dot plots at the top. As for METTL3, the most correlated pair is METTL3 – activated dendritic cells, the expression differences of METTL3 and activated dendritic cells in HCs and pSS is presented by dot plots at the bottom. *, p<0.05; **, p<0.01; ***, p<0.001.

Immune dysfunction plays a key role in the pathophysiology of pSS (27–30). The ssgsea analysis and the correlation analysis for DEMRs and immune characteristics were performed, to explore the involvement of m6A regulators in the immune microenvironment. Patients with pSS showed dysregulation of immune cell infiltrate, including dendritic cells (DCs), T cells, and CD56dim natural killer cells (**Figure 4A**). Decreased fractions of activated DCs and plasmacytoid DCs were found in pSS (*p* < 0.01), in accordance with the findings of Vogelsang et al. (28), which could be due to their migration from the circulatory system to the inflammatory tissues, including salivary glands and secondary lymphoid organs (29). Conversely, type 1, type 2, and type 17 T helper cells increased in pSS (**Figure 4A**). The correlation analysis revealed that m6A regulators were related to immunocytes, and among them, ALKBH5 and METTL3 showed a comprehensive linkage with the infiltrating immune cells (**Figures 4B-D** and **Supplementary Table 2**). METTL3 displayed the strongest negative correlation with the abundance of activated DCs and plasmacytoid DCs (r = -0.6, *p* < 0.001, **Figure 4D**), consistent with the findings that mRNA methylation mediated by METTL3 promoted DCs activation and T cell stimulation (5). These results indicated the key role of m6A regulation in mediating the immune dysfunction in pSS.

Additionally, the immune reaction score enriched by ssgsea revealed that natural killer cytotoxicity was decreased in pSS (**Supplementary Figure 2A**), which was consistent with our results of immune infiltration enrichment that showed a decrease in CD56dim natural killer cells in PB of pSS patients (**Figure 4A**). TCR signaling pathway and TNF family members receptors were also decreased in pSS (**Supplementary Figure 2A**). It is worth noting that METTL3 still showed the most significant correlation with the immune reaction gene-sets (**Supplementary Figure 2B**).

### FAA indicated the involvement of autophagy in pSS

**Figure 5:**
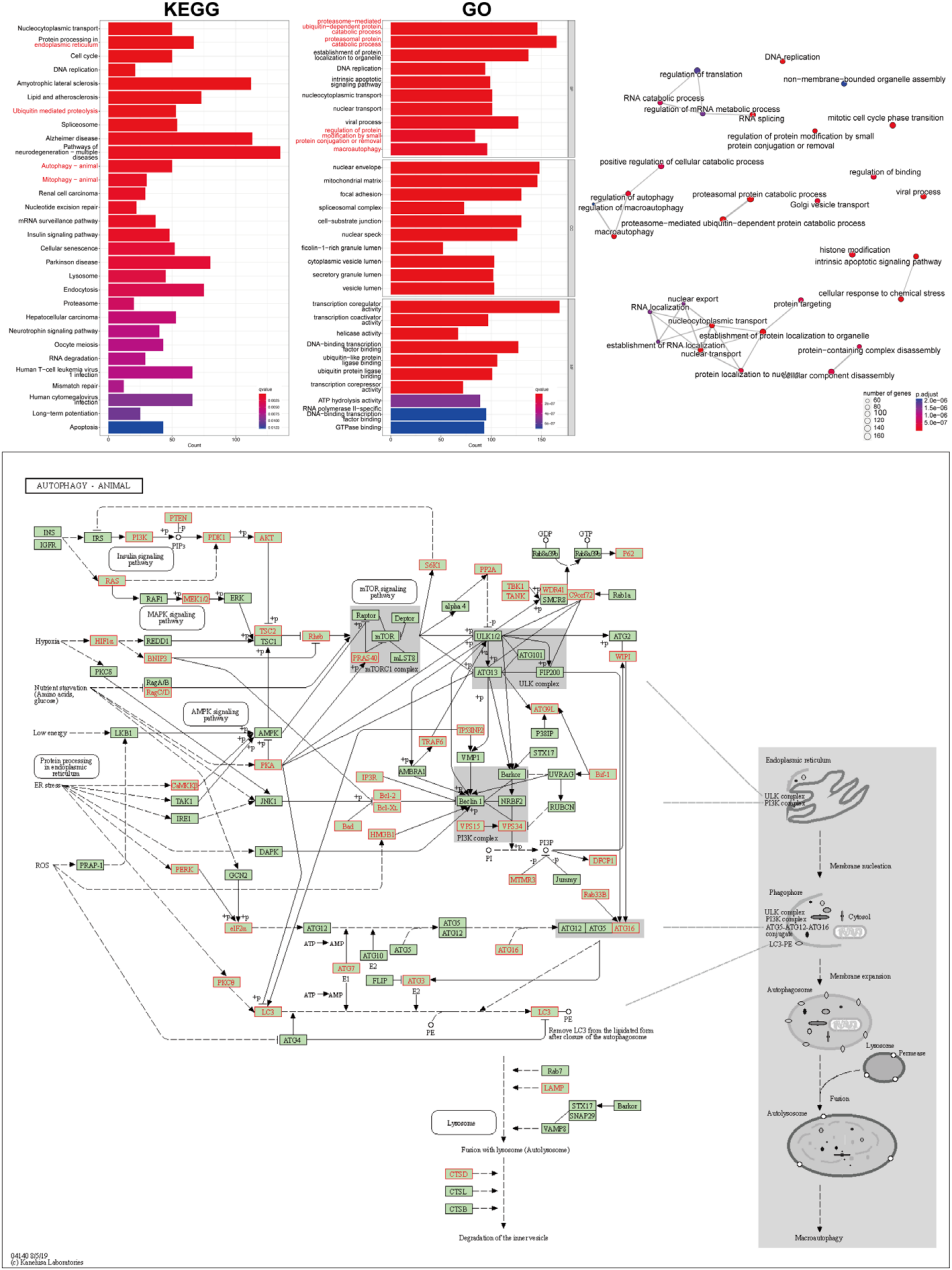
GO and KEGG pathway enrichment analysis on the genes mediated by key pSS-related m6A regulators. **(A)** The significantly enriched top 30 KEGG pathways. **(B)** The significantly enriched top 10 terms for GO: are BP, CC, and MF. **(C)** The emapplot visualizes gene sets of biological processes as a network, mutually overlapping gene sets tend to cluster together, making it easier for interpretation. Top 30 GO: BP terms were presented. **(D)** The “Autophagy-animal” pathway identified with KEGG enrichment.

The list of the target genes of DEMRs was obtained referring to the m6A2Target dataset. The co-expression analysis was also performed and the intersection of the two gene sets was defined as the genes mediated by DEMRs in our study, including 2071 genes regulated by METTL3, 1130 genes regulated by YTHDF1, 1090 genes regulated by ALKBH5, 293 genes regulated by RBM15B, 107 genes regulated by YTHDC2 and 1 gene regulated by RBMX. FAA was then performed on these genes mediated by DEMRs, and a list of significantly enriched pathways was identified (**Figure 5A-C**). Overall, the emapplot of GO : BP revealed three main biological mechanisms that included the most enriched terms emerging from the FAA analysis in pSS: RNA splicing, nuclear transport, and autophagy (**Figure 5C**).

Notably, the FAA results of KEGG and GO converged particularly to autophagy (**Figures 5A, B**), covering the most enriched KEGG terms including “protein processing in endoplasmic reticulum”, “ubiquitin mediated proteolysis”, “autophagy” and “mitophagy”, (**Figure 5A**) and GO : BP terms including “proteosome-mediated ubiquitin-dependent protein catabolic process”, “proteasomal protein catabolic process”, “regulation of protein modification by small protein conjugation or removal”, and “macroautophagy” (**Figure 5B**). The KEGG signaling pathway showed the comprehensive involvement of DEMR-mediated genes and their coaction in autophagy (**Figure 5D**, **Supplementary Figure 3A**). The cnetplot shows the gene names involved in terms related to KEGG_Autophagy and GO_Autophagy (**Supplementary Figures 3B, C**).

### Construction of the DEMR regulatory network for autophagy in pSS

**Figure 6:**
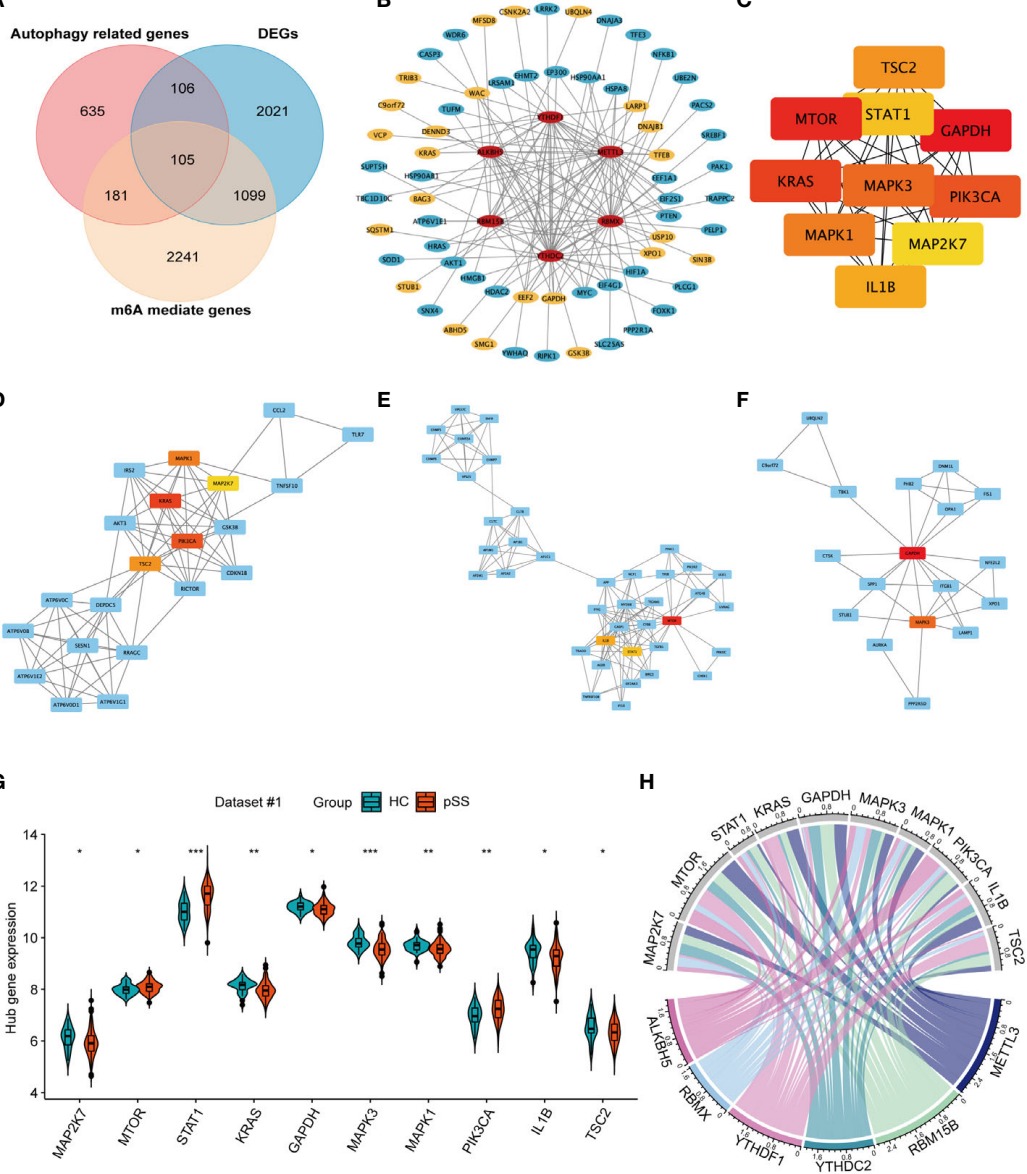
The network for autophagy-related genes modified by key pSS-related m6A regulators. **(A)** The venn plot illustrates the intersection of m6A mediated genes, autophagy-related genes, and the differentially expressed genes in pSS. **(B)** Protein-protein interaction (PPI) network among the pSS-related DEMRs and autophagy-related genes mediated by m6A regulators (n=286). Red represents m6A regulators, blue represents autophagy-related genes, among which the significantly expressed genes in pSS compared with HCs are highlighted in yellow. **(C)** The top 10 candidate hub genes of the differentially expressed autophagy-related genes (n=211) ranked by the maximum clique centrality (MCC) method. **(D-F)**. The top 3 subnetwork gene clusters were exported using the MCODE program. The hub genes were highlighted in red or yellow color as in **Figure 6C**. **(G)** The violin plot illustrates the autophagy hub genes that are dysregulated between the HCs and pSS groups in dataset #1. **(H)** The chord diagram demonstrated the correlation between DEMRs and autophagy hub genes. *, p<0.05; **, p<0.01; ***, p<0.001.

The Venn plot revealed the intersection of m6A-mediated genes, autophagy-related genes, and the differentially expressed genes (DEGs) in pSS (**Figure 6A**). Among the 3626 target genes of the 6 DEMRs, 286 were involved in autophagy. Further PPI network analysis showed the interaction among DEMRs and these autophagy-related genes (**Figure 6B**), revealing the regulatory role of m6A in autophagy. In addition, 211 autophagy-related genes that were dysregulated between pSS and HCs (**Figure 6A**) were uploaded into the PPI network analysis (**Figure S4**). The top 10 hub genes filtered by CytoHubba based on the MCC rank were identified (**Figure 6C**, **Supplementary Table 3**). The top 3 subnetwork gene clusters (cluster #1 with a score of 8.2; cluster #2 with a score of 2; cluster #3 with a score of 4.118) were exported using MCODE (**Figure 6D**). All hub genes fell in the three clusters, where MAPK1, MAP2K7, KRAS, PIK3CA, and TSC2 were located in cluster #1 (**Figure 6D**), MTOR, IL1B, and STAT1 2 were located in cluster #2 (**Figure 6E**) and GAPDH and MAPK3 2 were located in the cluster #3 (**Figure 6F**). PIK3CA, STAT1, and MAPK3 were located at the center of cluster #1, #2, and #3, respectively.

The expression of 10 autophagy hub genes in dataset #1 was compared between pSS and HCs (**Figure 6G**). The expression of MTOR, STAT1, and PIK3CA was up-regulated, while the other hub genes were downregulated. The chord diagram demonstrated the correlation between DEMRs and autophagy hub genes (**Figure 6H**), and the correlation coefficient r > 0.3 is shown in **Supplementary Table 4**. These results revealed the key regulation of the autophagy process by m6A in pSS.

### Validation of autophagy hub genes and the association with clinical features

**Figure 7:**
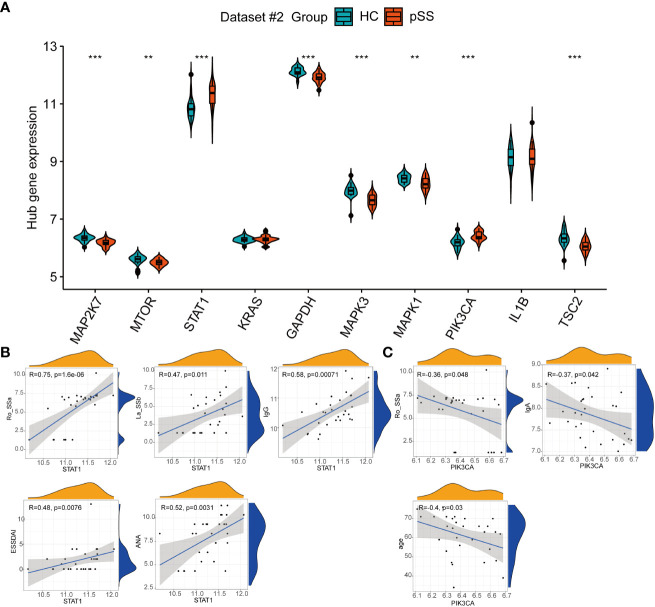
The expression of autophagy hub genes in pSS. **(A)** The violin plot illustrates the autophagy hub genes that are dysregulated between the HCs and pSS groups in dataset #2. **(B)** Correlations between STAT1 and laboratory tests in pSS patients in dataset #2. **(C)** Correlations between STAT1 and laboratory tests in pSS patients in dataset #2. **, p<0.01; ***, p<0.001.

Among the top 10 hub genes related to autophagy in pSS, the mRNA expression of STAT1 and PIK3CA in PB was significantly higher in pSS samples than in HC samples, whereas the mRNA expression of MAP2K7, MTOR, GAPDH, MAPK3, MAPK1, and TSC2 was higher in the HCs samples than in the pSS samples (**Figure 7A**).

The clinical features of 30 pSS patients in dataset #2 were downloaded. The diagnosis of pSS requires the presence of immunologic abnormalities, including ANA immunofluorescence, positive anti-SSA, and anti-SSB. The Spearman correlation analysis showed that the expression of STAT1 in PB showed a moderate to strong positive correlation with the immune response of pSS patients, including anti-SSA (r = 0.75, *p* < 0.001), anti-SSB (r = 0.47, *p* = 0.011), ANA (r = 0.52, *p* = 0.0031), and IgG (r = 0.58, *p* < 0.001), and associated with the disease activity based on the ESSDAI index (**Figure 7B**), in accordance to the results of Barrera et al (31). Conversely, PIK3CA showed a negative association with IgA, anti-SSA and age (**Figure 7C**).

### Heterogeneity related to anti-Ro/SSA antibody

Four m6A regulators in dataset #2 were identified as related to anti-Ro/SSA level or seroreactivity, including two writers (RBM15B and ZC3H13) and two readers (YTHDC1 and YTHDF1) (**Figure S5**). Compared with the group with low anti-Ro/SSA antibody levels, the B cells (activated B cells and immature B cells) of pSS patients in the group with high anti-Ro/SSA antibody levels showed higher infiltration (p<0.01) (**Supplementary Figure 6A**). However, this immune dysfunction was less correlated with m6A regulators (r=-0.38) (**Supplementary Figure 6B**). In addition, 122 genes were identified as differentially expressed between the pSS patients with high and low anti-SSA antibody levels, and they were dominantly involved in the defense response to viruses (**Supplementary Figure 6C**). Among them, 38.6% of genes (34/122) were mediated by the four m6A regulators related with anti-Ro/SSA antibodies and also mainly involved in the defense response to viruses and response to type I interferon (**Supplementary Figures 6D, E**).

### Verification of gene expression in our PB samples and labial salivary glands

**Figure 8:**
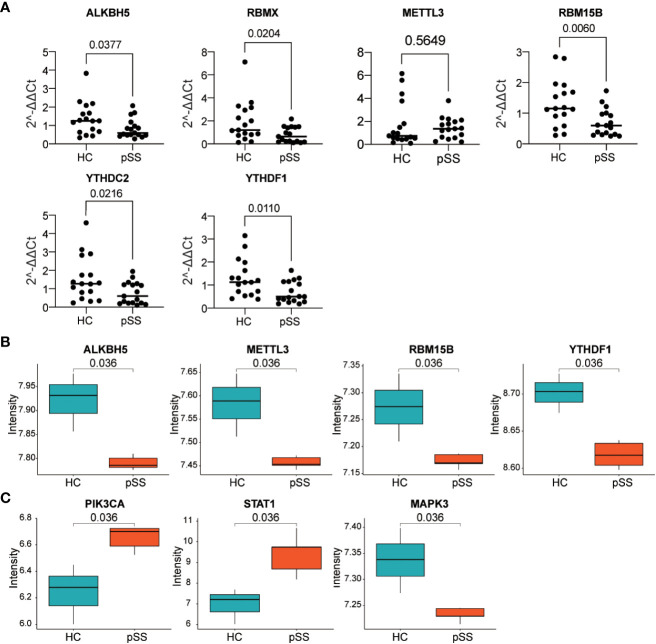
Verification of gene expression in our PB samples and labial salivary glands. **(A)** Verification of DEMRs in PB samples of our cohort. **(B, C)** Verification of DEMRs **(B)** and autophagy hub genes **(C)** in labial salivary glands of dataset #3.

The six DEMRs in the PUMCH PB cohort were validated. The mRNA expression of ALKBH5, RBMX, RBM15B, and YTHDF1 was significantly lower in pSS PB samples than that in HCs (**Figure 8A**). These results were consistent with those from dataset #1 and dataset #2. METTL3 mRNA expression was higher although not significant and YTHDC2 decreased significantly in pSS PB samples compared with that in HCs, results that were the opposite of those from the two GEO datasets of PB samples.

Considering the histopathology and the classification role of labial salivary glands in pSS, these findings, together with DEMRs, were further verified in samples from labial salivary gland biopsy of pSS and HCs in the dataset #3 (**Figure 8B**). RBMX was also not targeted in dataset #3, in which the expression profile was detected by Affymetrix GeneChip Human Genome U133 plus 2.0 Array as in dataset #2. Consistently, ALKBH5, METTL3, RBM15B, and YTHDF1 were downregulated in the tissue sample of pSS compared with those of HCs, whereas the upregulation of YTHDC2 was not verified in dataset #3. Notably, the hub genes PIK3CA, STAT1, and MAPK3 located at the center of the subnetwork cluster #1, #2, and #3 by MCODE respectively (**Figure 6**) were confirmed as significantly changed in the tissue samples of pSS than those in HCs (**Figure 8C**). Notably, the expression of 8,124 genes detected in dataset #1 and #3 showed a moderate correlation (r=0.455, *p*<0.0001, **Figure S7**), indicating that most genes maintained consistent changes between PB and labial salivary glands and their transcriptomes in pSS were overall moderately comparable, although inter-individual heterogeneity existed.

## Discussion

Recently, the epigenetic mechanism has been considered an emerging concern in human health and disease. It can alter gene expression without changing the DNA nucleotide sequence. Post-transcriptional modifications of mRNA, including m6A, 5-methylcytosine (m5C), and pseudouridine (ψ), are involved in the epigenetic regulation of multiple cellular processes, with broad roles in influencing mRNA stability, translation, and translocation (5, 6). The present study provided evidence that a peculiar profile of m6A regulators could distinguish pSS patients from HCs. The DEMRs identified from the array sequencing data were eventually validated in the other two GEO datasets of PB and in the samples of salivary gland tissue, as well as our collected fresh PB samples. Multiple immune reactions were disturbed in pSS, which could be modulated by m6A. Notably, the functional analysis of m6A mediated genes highlighted their relevance in the autophagy in pSS, hence suggesting the epigenetic regulation of m6A in the autophagy mechanisms in this disease.

m6A modification plays important roles in various cellular responses (5). The immune infiltration score for each subject was evaluated using ssgsea. The results revealed a significantly changed infiltration of the immunocyte profile including decreased activated DCs, plasmacytoid DCs, and CD56dim natural killer cells in pSS, in accordance with previous findings (27, 28). Since the cell infiltrates mainly consist of T cells and B cells, little attention has so far been directed to DCs. However, studies have uncovered the significance of DCs in pSS (28, 32). Plasmacytoid DCs circulate with PB and migrate to the inflamed tissues. Vogelsang et al. discovered that plasmacytoid DCs are decreased in PB, but their infiltration is detected in the salivary gland, implying their pathological role in the exocrinopathy of pSS patients (28). Besides the altered population and enumeration of DCs in PB (28), the activation of plasmacytoid DCs converges with the increased type-I IFN production and enhanced survival (32). The current study first proposes that m6A may modulate DCs in pSS, which has been previously reported in cancer, since m6A marked mRNAs encoding lysosomal proteases recognized by m6A “readers” can suppress the antigen presentation and promote immune evasion (33). Our results on METTL3 with the strongest negative correlation with the immune infiltration score of DCs, are remarkably consistent with those of Wang et al. who revealed that the specific depletion of METTL3 in DCs results in the impaired activation of DCs, with a decreased expression of the co-stimulatory molecules CD40, CD80 and IL-12, and reduced T cell responses (5). The immune score of other immunocytes was also identified, including CD56dim natural killer cells, T helper cells and monocytes, and their close association with m6A regulators further provided insight to the m6A epigenetic regulation on the immune responses in pSS. Interestingly, heterogeneity within pSS patients existed, and the abunt B cell infiltration (activated B cells and immature B cells) was identified related to the higher levels anti-Ro/SSA antibody.

The FAA on the m6A mediated genes derived from the database and co-expression results was performed to further illustrate the downstream molecular mechanisms and functions of DEMRs related to pSS. In particular, the most innovative finding emerging from the FAA results confirmed the undebated role of m6A methylation on autophagy in pSS, consistent with previous study on abdominal aortic aneurysm (12) and epithelial ovarian cancer (34). Autophagy is an evolutionarily conserved mechanism, which widely exists in eukaryotes, and includes at least three types: macroautophagy, microautophagy, and chaperone-mediated autophagy (35). It promotes cell survival and the homeostatic state in harsh conditions by breaking down dysfunctional or unnecessary organelles and proteins, or microorganisms such as viruses and bacteria (35, 36). It is accepted that autophagy or the unique functions of autophagic proteins may serve as a central fulcrum to balance the beneficial and harmful effects of the host’s response to infections and other immunological stimuli (36). Altered autophagy or autophagic protein function results in maladaptive inflammatory and metabolic responses, therefore causing the development of more severe diseases (36). Various pathophysiological conditions have identified different m6A modifications regulating aberrant autophagy (34, 37–39).

The pathologic role of autophagy in the lymphocyte infiltration of exocrine glands, as well as in the survival and activation of epithelial cells in the salivary gland has previously been described in pSS (3). Colafrancesco et al (3, 40, 41) demonstrated that up-regulated autophagy pathways induced by inflammation activate salivary gland epithelial cells in pSS, causing the high expression of adhesion molecules, and it is associated with histologic severity. This study discovered hub genes of autophagy in pSS, including PIK3CA, STAT1, MAPK3, and MTOR. Since the activated epithelial cells of the salivary glands could be the initiating factor causing immune and inflammatory disorders and the dryness symptoms in pSS, their expression in salivary gland tissues was further verified, which was closely interlinked to the altered expression of m6A regulators. Consistently, the modulation of m6A on autophagy has been previously reported. Liang et al. unveiled that the m6A reader YTHDC1 modulates autophagy in diabetic keratinocytes through the regulation of the stability of the nuclear sequestosome 1 (SQSTM1) mRNA (an autophagy receptor) (37). The depletion of METTL3, a primary m6A methyltransferase, under hypoxia activates autophagy-associated pathways in cultured hepatocellular carcinoma cells (38). As regards metabolic diseases, METLL3 and the partner YTHDF1 promote the hepatic autophagic flux and the clearance of lipid droplets in nonalcoholic fatty liver disease (39). PI3K-AKT and MAPK-ERK are upstream signals of mTOR, which activate TOR and consequently inhibit autophagy. ALKBH5 regulates autophagy through the mTOR pathway. Silencing the ALKBH5 inhibits p-AKT, p-MAPK, and p-ERK, while the ectopic expression of ALKBH5 promotes its expression (34).

Discrepancies were observed between different datasets and several potential factors should be considered. First, the clinical background, including the disease severity and activity of patients, was different among the cohorts of these datasets, which greatly affects the dysregulation of m6A regulators (19). Second, the subjects in different datasets had different treatment histories and the treatment received may affect the expression of m6A regulators (19, 42). For example, pSS patients in dataset #1 who were treated with moderate to high doses of corticosteroids, immunosuppressants, or biological agents were excluded, and HCs were those who did not receive any drug. However, all patients and HCs in dataset #3 were naive to immunosuppressive therapy. Third, different detection platforms were applied to perform the high-throughput sequencing.

This study presents some limitations. First, the molecular mechanisms underlying the influence of m6A modification on pSS should be explored by *in vivo* and *in vitro* experiments for epigenetic-based therapy. Second, the further exploration of the interaction between m6A and autophagy in the gland tissues and the difference in the expression pattern with PB was hampered due to the limited cohorts in dataset #3. Third, the expression of DEMRs and autophagy hub genes in dataset #3 should be further verified considering the key role of the epithelial cells of the salivary gland in the pathogenesis of pSS. However, the samples from dataset #3 were entirely salivary gland tissues rather than epithelial cells isolated from the primary salivary gland and were contaminated with lymphocytes, potentially compromising the observation (31). Further studies recruiting a larger cohort with PB samples and exocrine gland epithelial tissue samples should be performed to discover the pathological mechanisms of DEMRs in the autophagy involved in pSS.

## Conclusion

To date, this study represents the first prospective study on PB and exocrine glands from pSS patients in the investigation of the m6A epigenetic mechanism. The crosstalk between m6A epigenetic modification and the remarkable up-regulation of autophagy pathways in pSS was revealed, which acted as a contributor to the pathogenesis of pSS and represents a novel therapeutic target. Despite the available biological agents and disease-modifying antirheumatic drugs for nearly all rheumatic diseases, firm evidence should be acquired to recommend drugs for pSS (1). The present findings together with previous studies on autophagy in pSS strongly suggest the therapeutic potential of innovative alternative strategies disrupting this mechanistic connection through m6A modification.

## Data availability statement

The original contributions presented in the study are included in the article/**Supplementary Material**. Further inquiries can be directed to the corresponding author.

## Ethics statement

The studies involving human participants were reviewed and approved by the Medical Ethics Committee of Peking Union Medical College Hospital (JS-2049). The patients/participants provided their written informed consent to participate in this study.

## Author contributions

YZL conceived and designed the research. LLC extracted data, performed software analysis and visualized graphs and tables. LLC, HLL, and HTZ went on validation experiments. LLC wrote the paper and HTZ proofread the paper. YZL, XML, and YH categorized and supervised graphs and tables. LW and FCZ provided the samples and the clinical information. All authors contributed to the article and approved the submitted version.

## Funding

This work was supported by the National Key Research and Development Program of China (2018YFE0207300), the National Natural Science Foundation of China (81871302), and Beijing Key Clinical Specialty for Laboratory Medicine - Excellent Project (No. ZK201000)

## Conflict of interest

The authors declare that the research was conducted in the absence of any commercial or financial relationships that could be construed as a potential conflict of interest.

## Publisher’s note

All claims expressed in this article are solely those of the authors and do not necessarily represent those of their affiliated organizations, or those of the publisher, the editors and the reviewers. Any product that may be evaluated in this article, or claim that may be made by its manufacturer, is not guaranteed or endorsed by the publisher.
