# Supplementary material for: Alterations of m6A RNA methylation regulators contribute to autophagy and immune infiltration in primary Sjögren’s syndrome
Source: Front Immunol. 2022 Sep 20;13:949206. doi: 10.3389/fimmu.2022.949206 (PMC9530814; doi:10.3389/fimmu.2022.949206)
Supplement: Supplementary file 1 [file DataSheet_1.docx]

**-Supplementary materials-**

**4 Supplementary Tables**

| Table S1 | Demographic and clinical data of the PUMCH cohort |
| --- | --- |
| Table S2 | Correlations between m6A regulators and immunocytes in pSS |
| Table S3 | The top 10 hub genes of the differentially expressed autophagy related genes ranked by maximum clique centrality (MCC) method |
| Table S4 | The correlation between DEMRs and autophagy hub genes |

**Table S1 Demographic and clinical data of the PUMCH cohort.**

| HCs | | | | pSS | | | | | | | | | | | | | | | | |
| --- | --- | --- | --- | --- | --- | --- | --- | --- | --- | --- | --- | --- | --- | --- | --- | --- | --- | --- | --- | --- |
| ID | Date | Age | Gender | ID | Date | Age | Gender | CRP | IgG | IgA | IgM | ESR | C3 | C4 | ANA | anti-dsDNA | anti-SSA | anti-SSB | anti-RO52 | RF |
| HC1 | 2022.4.7 | 37 | Female | pSS1 | 2022.3.17 | 40 | Female | 0.14 | 18.64 | 3.4 | 2.15 | 5 | 0.713 | 0.12 | + | - | + | - | + | / |
| HC2 | 2022.4.8 | 45 | Female | pSS2 | 2022.3.18 | 47 | Female | 1.1 | 20.18 | 3.54 | 3.28 | 20 | / | / | / | / | / | / | / | 339.4 |
| HC3 | 2022.3.22 | 48 | Female | pSS3 | 2022.3.18 | 49 | Female | 4.66 | 15.16 | 4.07 | 0.27 | 60 | 1.461 | 0.383 | + | - | + | + | + | / |
| HC4 | 2022.3.22 | 37 | Female | pSS4 | 2022.3.18 | 70 | Female | 1.21 | / | / | / | / | / | / | + | - | + | - | - | / |
| HC5 | 2022.3.22 | 60 | Female | pSS5 | 2022.3.24 | 60 | Female | 0.84 | 20.55 | 4.29 | 1.26 | 26 | / | / | + | - | + | + | + | 425.2 |
| HC7 | 2022.3.28 | 27 | Female | pSS7 | 2022.3.28 | 26 | Female | 0.36 | 11.92 | 2.47 | 1.48 | 3 | 1.028 | 0.123 | + | - | - | + | - | / |
| HC6 | 2022.4.8 | 49 | Female | pSS6 | 2022.3.28 | 53 | Female | 0.56 | 18.3 | 5.55 | 0.82 | 11 | 1.16 | 0.207 | + | - | + | + | + | / |
| HC8 | 2022.4.2 | 31 | Female | pSS8 | 2022.4.1 | 32 | Female | 0.18 | 23.31 | 1.95 | 2.67 | 5 | 0.75 | 0.089 | + | + | + | - | + | / |
| HC9 | 2022.4.7 | 33 | Female | pSS9 | 2022.4.2 | 35 | Female | / | 16.02 | 3.77 | 0.25 | / | 0.903 | 0.228 | + | - | + | - | + | 14.9 |
| HC10 | 2022.4.7 | 39 | Female | pSS10 | 2022.4.2 | 42 | Female | 1.65 | 19.49 | 2.7 | 1.67 | 7 | 0.849 | 0.196 | + | - | + | - | + | / |
| HC11 | 2022.4.8 | 44 | Female | pSS11 | 2022.4.2 | 46 | Female | 2.06 | / | / | / | / | / | / | + | - | + | + | + | / |
| HC12 | 2022.3.28 | 28 | Female | pSS12 | 2022.4.3 | 32 | Female | <0.5 | 18.4 | 2.28 | 1.41 | 13 | 0.83 | 0.151 | + | - | + | + | + | 136.6 |
| HC13 | 2022.4.8 | 45 | Female | pSS13 | 2022.4.6 | 73 | Female | 1.25 | 20.52 | 2.52 | 1.47 | 53 | 0.71 | 0.144 | + | - | + | + | + | / |
| HC14 | 2022.3.18 | 54 | Female | pSS14 | 2022.4.6 | 56 | Female | 1.85 | 10.2 | 1.63 | 0.59 | 20 | 1.24 | 0.309 | + | - | + | - | + | 7.8 |
| HC15 | 2022.3.28 | 35 | Female | pSS15 | 2022.4.8 | 36 | Female | 0.87 | 12.7 | 2.41 | 0.97 | 7 | 0.995 | 0.244 | + | - | / | / | / | / |
| HC16 | 2022.3.24 | 61 | Female | pSS16 | 2022.4.8 | 60 | Female | 0.91 | 9.61 | 8.82 | 1.17 | 18 | 0.791 | 0.187 | + | - | + | - | - | 7.3 |
| HC17 | 2022.3.22 | 48 | Female | pSS17 | 2022.4.8 | 56 | Female | 0.43 | 17.54 | 4.38 | 1.11 | 27 | 1.04 | 0.151 | + | - | + | - | + | / |

**Table S2 Correlations between m6A regulators and immunocytes in pSS**

| m6A regulators | immune cells | r | p |
| --- | --- | --- | --- |
| METTL3 | Activated.B.cell | 0.18 | 0.01 |
| RBM15B | Activated.B.cell | -0.17 | 0.02 |
| YTHDC2 | Activated.B.cell | 0.02 | 0.77 |
| YTHDF1 | Activated.B.cell | -0.05 | 0.50 |
| RBMX | Activated.B.cell | 0.22 | 0.00 |
| ALKBH5 | Activated.B.cell | 0.14 | 0.06 |
| METTL3 | Activated.CD4.T.cell | 0.34 | 0.00 |
| RBM15B | Activated.CD4.T.cell | -0.14 | 0.06 |
| YTHDC2 | Activated.CD4.T.cell | 0.49 | 0.00 |
| YTHDF1 | Activated.CD4.T.cell | -0.16 | 0.03 |
| RBMX | Activated.CD4.T.cell | 0.32 | 0.00 |
| ALKBH5 | Activated.CD4.T.cell | 0.14 | 0.06 |
| METTL3 | Activated.CD8.T.cell | 0.07 | 0.32 |
| RBM15B | Activated.CD8.T.cell | -0.02 | 0.82 |
| YTHDC2 | Activated.CD8.T.cell | 0.10 | 0.17 |
| YTHDF1 | Activated.CD8.T.cell | 0.02 | 0.82 |
| RBMX | Activated.CD8.T.cell | 0.15 | 0.04 |
| ALKBH5 | Activated.CD8.T.cell | 0.10 | 0.16 |
| METTL3 | Activated.dendritic.cell | -0.60 | 0.00 |
| RBM15B | Activated.dendritic.cell | -0.04 | 0.58 |
| YTHDC2 | Activated.dendritic.cell | -0.38 | 0.00 |
| YTHDF1 | Activated.dendritic.cell | -0.35 | 0.00 |
| RBMX | Activated.dendritic.cell | -0.22 | 0.00 |
| ALKBH5 | Activated.dendritic.cell | -0.37 | 0.00 |
| METTL3 | CD56bright.natural.killer.cell | -0.11 | 0.14 |
| RBM15B | CD56bright.natural.killer.cell | 0.21 | 0.00 |
| YTHDC2 | CD56bright.natural.killer.cell | -0.07 | 0.34 |
| YTHDF1 | CD56bright.natural.killer.cell | 0.15 | 0.04 |
| RBMX | CD56bright.natural.killer.cell | -0.09 | 0.24 |
| ALKBH5 | CD56bright.natural.killer.cell | 0.16 | 0.03 |
| METTL3 | CD56dim.natural.killer.cell | -0.09 | 0.20 |
| RBM15B | CD56dim.natural.killer.cell | 0.34 | 0.00 |
| YTHDC2 | CD56dim.natural.killer.cell | -0.18 | 0.01 |
| YTHDF1 | CD56dim.natural.killer.cell | -0.03 | 0.66 |
| RBMX | CD56dim.natural.killer.cell | 0.30 | 0.00 |
| ALKBH5 | CD56dim.natural.killer.cell | 0.53 | 0.00 |
| METTL3 | Eosinophil | -0.28 | 0.00 |
| RBM15B | Eosinophil | -0.10 | 0.16 |
| YTHDC2 | Eosinophil | -0.12 | 0.10 |
| YTHDF1 | Eosinophil | -0.23 | 0.00 |
| RBMX | Eosinophil | -0.18 | 0.01 |
| ALKBH5 | Eosinophil | -0.25 | 0.00 |
| METTL3 | Gamma.delta.T.cell | -0.20 | 0.01 |
| RBM15B | Gamma.delta.T.cell | -0.01 | 0.87 |
| YTHDC2 | Gamma.delta.T.cell | 0.02 | 0.81 |
| YTHDF1 | Gamma.delta.T.cell | -0.33 | 0.00 |
| RBMX | Gamma.delta.T.cell | 0.10 | 0.18 |
| ALKBH5 | Gamma.delta.T.cell | -0.10 | 0.15 |
| METTL3 | Immature..B.cell | -0.01 | 0.85 |
| RBM15B | Immature..B.cell | 0.00 | 0.95 |
| YTHDC2 | Immature..B.cell | -0.12 | 0.10 |
| YTHDF1 | Immature..B.cell | -0.15 | 0.04 |
| RBMX | Immature..B.cell | 0.19 | 0.01 |
| ALKBH5 | Immature..B.cell | 0.10 | 0.16 |
| METTL3 | Immature.dendritic.cell | -0.01 | 0.90 |
| RBM15B | Immature.dendritic.cell | -0.16 | 0.03 |
| YTHDC2 | Immature.dendritic.cell | -0.05 | 0.52 |
| YTHDF1 | Immature.dendritic.cell | -0.07 | 0.37 |
| RBMX | Immature.dendritic.cell | 0.01 | 0.88 |
| ALKBH5 | Immature.dendritic.cell | -0.05 | 0.53 |
| METTL3 | MDSC | -0.18 | 0.01 |
| RBM15B | MDSC | 0.22 | 0.00 |
| YTHDC2 | MDSC | -0.14 | 0.05 |
| YTHDF1 | MDSC | -0.14 | 0.05 |
| RBMX | MDSC | 0.11 | 0.12 |
| ALKBH5 | MDSC | 0.29 | 0.00 |
| METTL3 | Macrophage | -0.27 | 0.00 |
| RBM15B | Macrophage | -0.03 | 0.65 |
| YTHDC2 | Macrophage | -0.21 | 0.00 |
| YTHDF1 | Macrophage | -0.14 | 0.06 |
| RBMX | Macrophage | -0.27 | 0.00 |
| ALKBH5 | Macrophage | -0.29 | 0.00 |
| METTL3 | Mast.cell | -0.23 | 0.00 |
| RBM15B | Mast.cell | -0.07 | 0.37 |
| YTHDC2 | Mast.cell | -0.16 | 0.03 |
| YTHDF1 | Mast.cell | -0.19 | 0.01 |
| RBMX | Mast.cell | -0.20 | 0.01 |
| ALKBH5 | Mast.cell | -0.22 | 0.00 |
| METTL3 | Monocyte | -0.42 | 0.00 |
| RBM15B | Monocyte | 0.41 | 0.00 |
| YTHDC2 | Monocyte | -0.39 | 0.00 |
| YTHDF1 | Monocyte | -0.13 | 0.08 |
| RBMX | Monocyte | 0.10 | 0.18 |
| ALKBH5 | Monocyte | 0.28 | 0.00 |
| METTL3 | Natural.killer.T.cell | 0.02 | 0.82 |
| RBM15B | Natural.killer.T.cell | 0.24 | 0.00 |
| YTHDC2 | Natural.killer.T.cell | -0.01 | 0.89 |
| YTHDF1 | Natural.killer.T.cell | 0.12 | 0.10 |
| RBMX | Natural.killer.T.cell | 0.02 | 0.82 |
| ALKBH5 | Natural.killer.T.cell | 0.20 | 0.00 |
| METTL3 | Natural.killer.cell | -0.35 | 0.00 |
| RBM15B | Natural.killer.cell | -0.17 | 0.02 |
| YTHDC2 | Natural.killer.cell | -0.20 | 0.01 |
| YTHDF1 | Natural.killer.cell | -0.08 | 0.30 |
| RBMX | Natural.killer.cell | -0.37 | 0.00 |
| ALKBH5 | Natural.killer.cell | -0.45 | 0.00 |
| METTL3 | Neutrophil | -0.31 | 0.00 |
| RBM15B | Neutrophil | -0.13 | 0.06 |
| YTHDC2 | Neutrophil | -0.12 | 0.10 |
| YTHDF1 | Neutrophil | -0.09 | 0.20 |
| RBMX | Neutrophil | -0.39 | 0.00 |
| ALKBH5 | Neutrophil | -0.48 | 0.00 |
| METTL3 | Plasmacytoid.dendritic.cell | -0.39 | 0.00 |
| RBM15B | Plasmacytoid.dendritic.cell | 0.00 | 0.97 |
| YTHDC2 | Plasmacytoid.dendritic.cell | -0.23 | 0.00 |
| YTHDF1 | Plasmacytoid.dendritic.cell | -0.02 | 0.79 |
| RBMX | Plasmacytoid.dendritic.cell | -0.34 | 0.00 |
| ALKBH5 | Plasmacytoid.dendritic.cell | -0.39 | 0.00 |
| METTL3 | Regulatory.T.cell | 0.16 | 0.03 |
| RBM15B | Regulatory.T.cell | -0.47 | 0.00 |
| YTHDC2 | Regulatory.T.cell | 0.17 | 0.02 |
| YTHDF1 | Regulatory.T.cell | -0.26 | 0.00 |
| RBMX | Regulatory.T.cell | -0.08 | 0.25 |
| ALKBH5 | Regulatory.T.cell | -0.35 | 0.00 |
| METTL3 | T.follicular.helper.cell | -0.07 | 0.31 |
| RBM15B | T.follicular.helper.cell | 0.34 | 0.00 |
| YTHDC2 | T.follicular.helper.cell | -0.15 | 0.05 |
| YTHDF1 | T.follicular.helper.cell | 0.09 | 0.21 |
| RBMX | T.follicular.helper.cell | 0.07 | 0.32 |
| ALKBH5 | T.follicular.helper.cell | 0.18 | 0.01 |
| METTL3 | Type.1.T.helper.cell | -0.35 | 0.00 |
| RBM15B | Type.1.T.helper.cell | -0.09 | 0.21 |
| YTHDC2 | Type.1.T.helper.cell | -0.21 | 0.00 |
| YTHDF1 | Type.1.T.helper.cell | -0.12 | 0.11 |
| RBMX | Type.1.T.helper.cell | -0.20 | 0.01 |
| ALKBH5 | Type.1.T.helper.cell | -0.24 | 0.00 |
| METTL3 | Type.17.T.helper.cell | -0.21 | 0.00 |
| RBM15B | Type.17.T.helper.cell | 0.04 | 0.55 |
| YTHDC2 | Type.17.T.helper.cell | -0.16 | 0.03 |
| YTHDF1 | Type.17.T.helper.cell | -0.10 | 0.19 |
| RBMX | Type.17.T.helper.cell | -0.23 | 0.00 |
| ALKBH5 | Type.17.T.helper.cell | -0.34 | 0.00 |
| METTL3 | Type.2.T.helper.cell | 0.16 | 0.03 |
| RBM15B | Type.2.T.helper.cell | -0.12 | 0.10 |
| YTHDC2 | Type.2.T.helper.cell | 0.16 | 0.02 |
| YTHDF1 | Type.2.T.helper.cell | 0.04 | 0.58 |
| RBMX | Type.2.T.helper.cell | -0.20 | 0.01 |
| ALKBH5 | Type.2.T.helper.cell | 0.03 | 0.64 |

**Table S3 The top 10 hub genes of the differentially expressed autophagy related genes ranked by maximum clique centrality (MCC) method**

| Rank | Name | Score |
| --- | --- | --- |
| 1 | GAPDH | 945934 |
| 2 | MTOR | 792242 |
| 3 | KRAS | 668083 |
| 4 | PIK3CA | 620286 |
| 5 | MAPK3 | 549352 |
| 6 | MAPK1 | 508322 |
| 7 | TSC2 | 492302 |
| 8 | IL1B | 458582 |
| 9 | STAT1 | 400602 |
| 10 | MAP2K7 | 330030 |

**Table S4 The correlation between DEMRs and autophagy hub genes**

| m6A regulators | autophagy genes | r | p |
| --- | --- | --- | --- |
| METTL3 | MTOR | 0.53898763 | 1.04E-15 |
| METTL3 | KRAS | -0.3810223 | 5.85E-08 |
| METTL3 | GAPDH | -0.3922809 | 2.17E-08 |
| METTL3 | IL1B | -0.4092933 | 4.54E-09 |
| RBM15B | MTOR | 0.37174302 | 1.29E-07 |
| RBM15B | GAPDH | 0.44086591 | 1.94E-10 |
| RBM15B | PIK3CA | -0.4240249 | 1.09E-09 |
| RBM15B | TSC2 | 0.35266775 | 6.03E-07 |
| YTHDC2 | MAP2K7 | -0.3478891 | 8.74E-07 |
| YTHDC2 | MTOR | 0.3102764 | 1.32E-05 |
| YTHDC2 | GAPDH | -0.3625661 | 2.74E-07 |
| YTHDC2 | MAPK3 | -0.3106722 | 1.29E-05 |
| YTHDF1 | MTOR | 0.52520645 | 7.29E-15 |
| YTHDF1 | KRAS | -0.4082675 | 5.00E-09 |
| YTHDF1 | TSC2 | 0.4910716 | 6.32E-13 |
| RBMX | MAPK3 | -0.3178542 | 7.86E-06 |
| ALKBH5 | MTOR | 0.44441694 | 1.33E-10 |
| ALKBH5 | PIK3CA | -0.5320412 | 2.80E-15 |

**7 Supplementary Figures**

| Figure S1 | Nomogram based on DEMRs |
| --- | --- |
| Figure S2 | The immune reactions characteristics in pSS |
| Figure S3 | The autophagy terms by functional annotation analysis and related genes |
| Figure S4 | The protein–protein interaction network of the 211 autophagy related genes that differentially expressed between pSS and healthy controls |
| Figure S5 | The differentially expressed genes that related to anti-Ro/SSA. |
| Figure S6 | Distinct biological characteristics related to anti-Ro/SSA seroreactivity |
| Figure S7 | Scatter plot demonstrating the correlation in gene expression between peripheral blood (dataset #1) and labial salivary glands (dataset #3). |

**
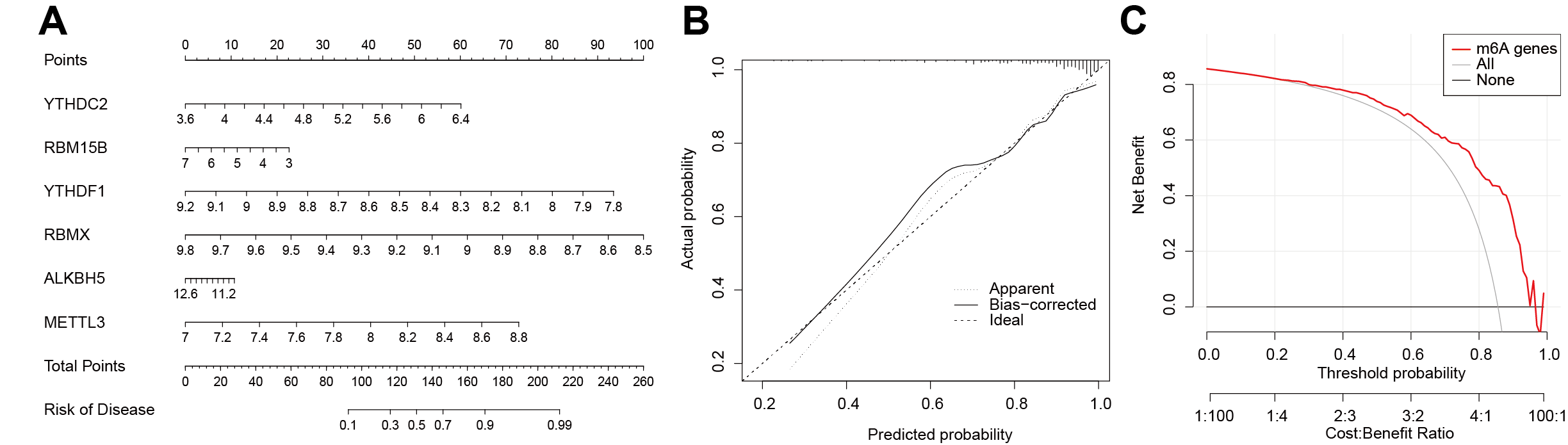
**

**Figure S1 Nomogram based on DEMRs.** A. The nomogram by 6 DEMRs was constructed. B. The calibration chart showing the bias-corrected line and the ideal curve. C. Decision curve analyses (DCA) for assessing clinical predictive models.


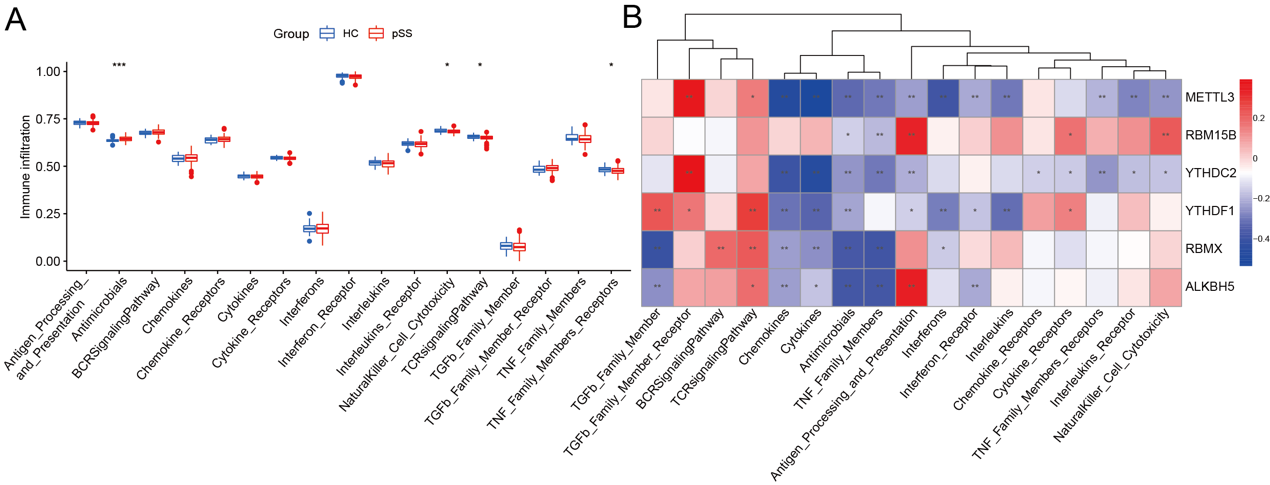


**Figure S2 The immune reactions characteristics in pSS.** A. The differences of the immune reaction score in HCs and pSS. B. The correlation analysis between immune reaction score and the 6 DEMRs.


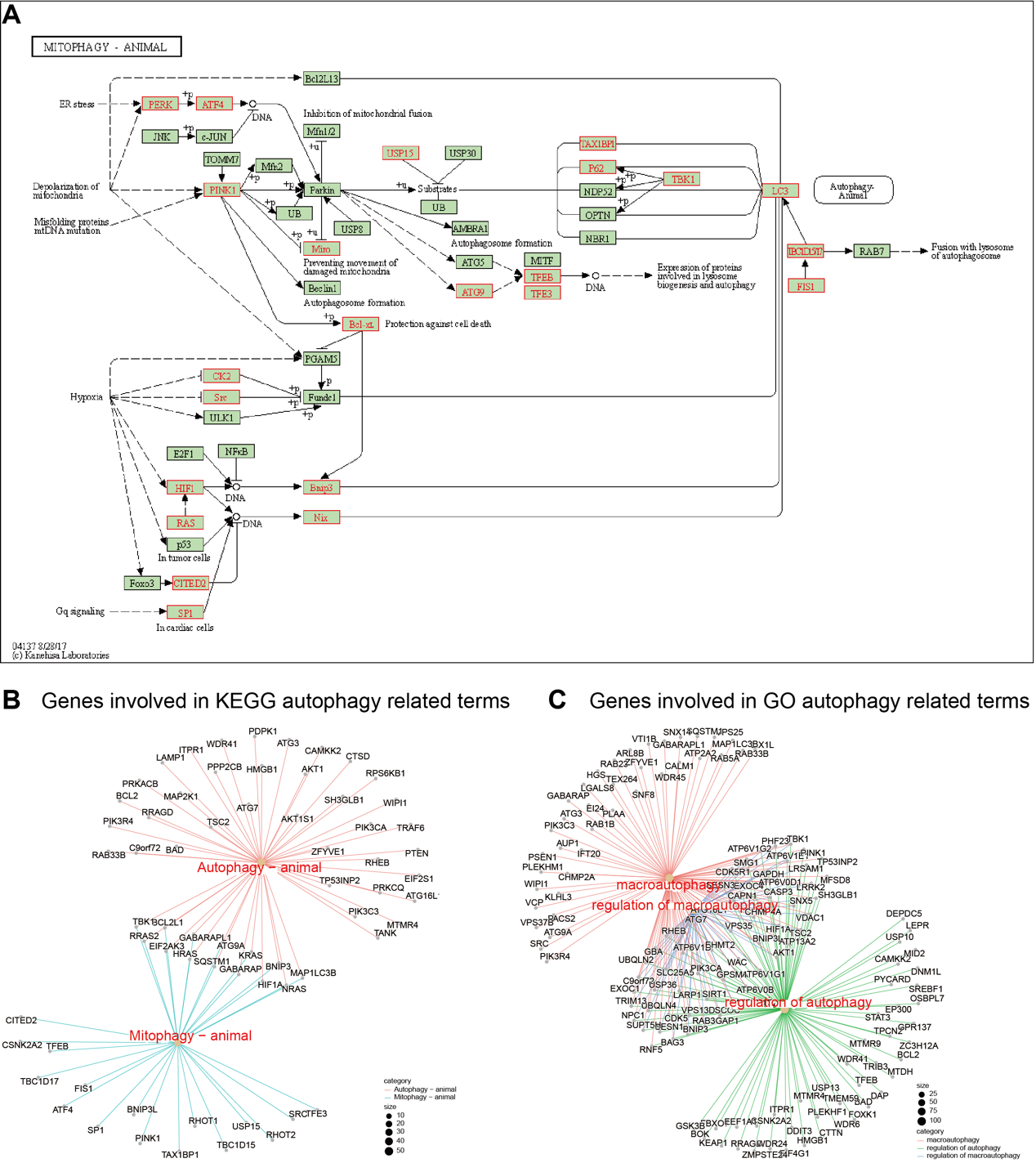


**Figure S3 The autophagy terms by functional annotation analysis.** A. The “Mitophagy-animal” pathway identified with KEGG enrichment. B.-C. The cnetplots illustrate the gene names involved in terms related to KEGG_Autophagy (B) and GO_Autophagy (C).


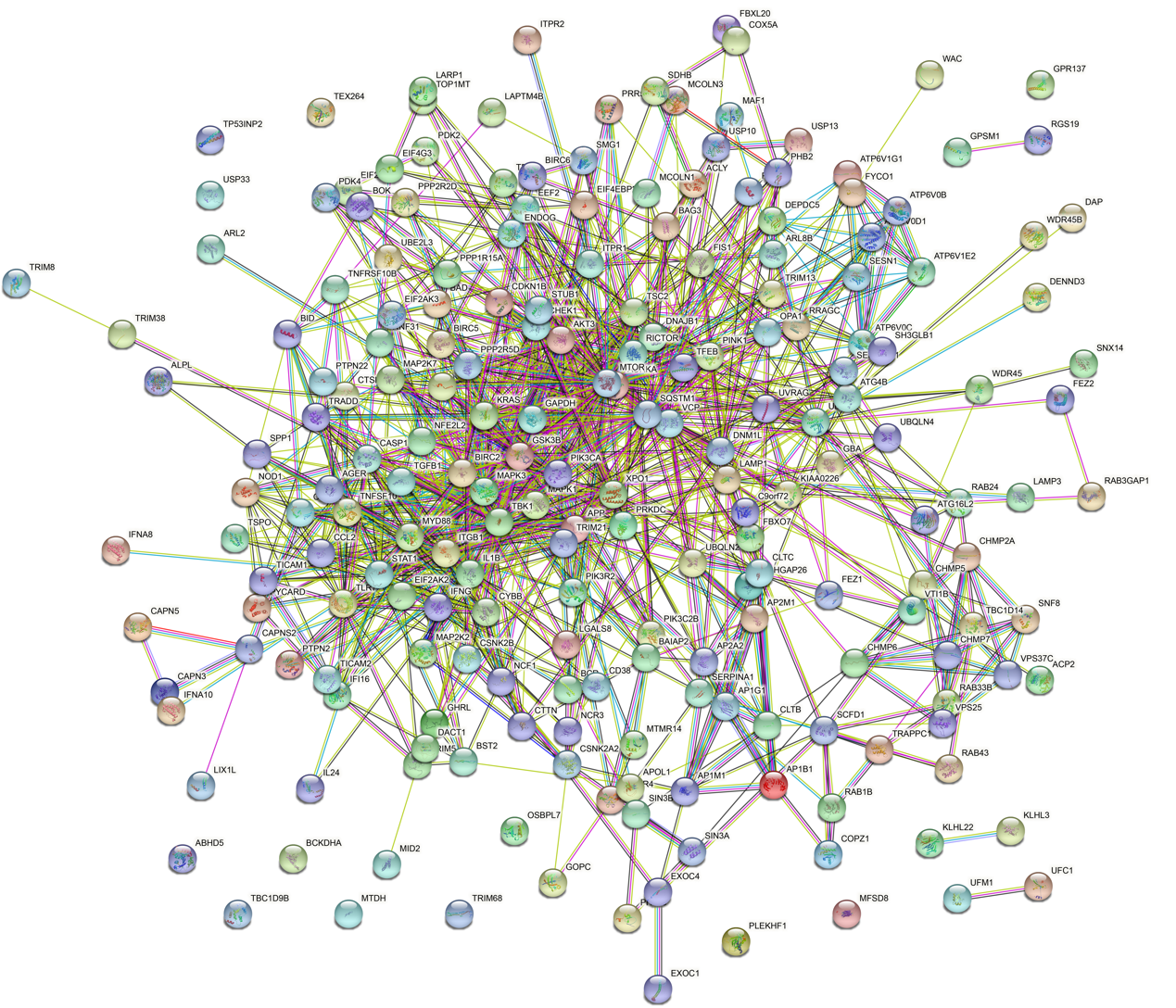


**Figure S4 The protein–protein interaction network of the 211 autophagy related genes that differentially expressed between pSS and healthy controls.**

**
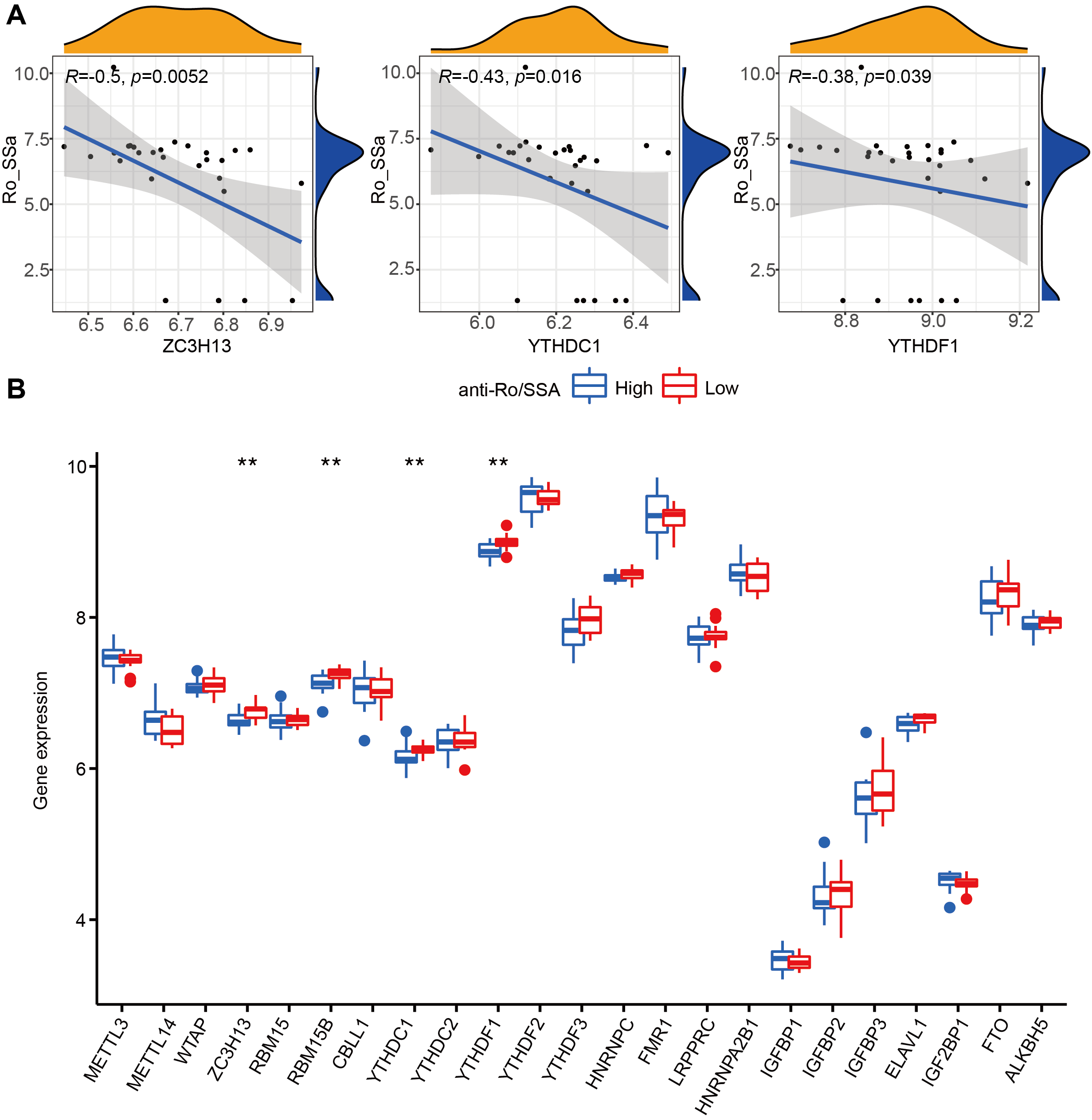
**

**Figure S5 The differentially expressed genes that related to anti-Ro/SSA.** A. Correlations between the expression of m6A regulators and anti-Ro/SSA level in pSS patients in dataset #3. B. The box plot demonstrated the transcriptome expression status of the m6A regulators between groups of pSS patients with high and low anti-Ro/SSA antibody level in dataset #3.

**
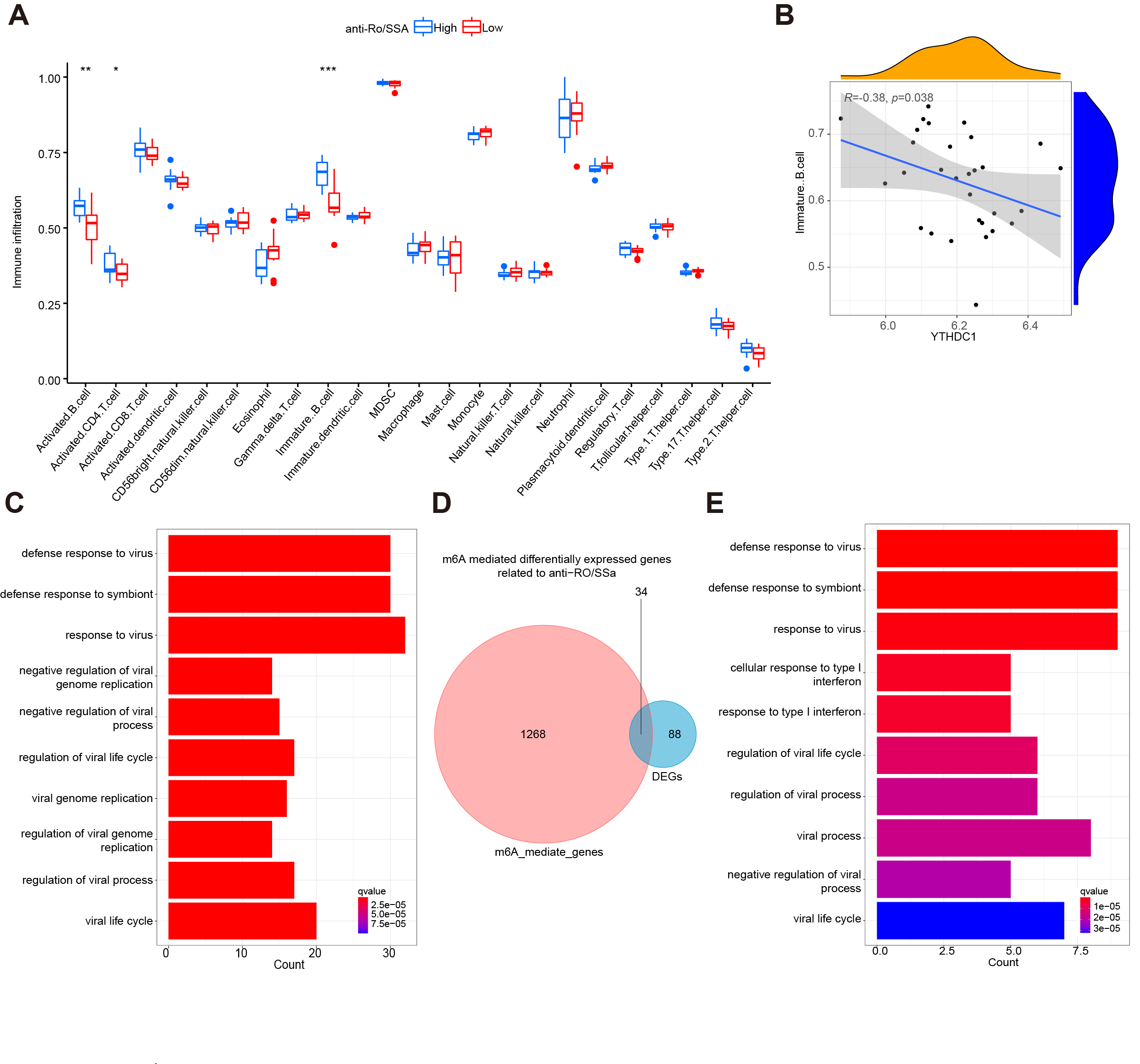
**

**Figure S6 Distinct biological characteristics related to anti-Ro/SSA sero-reactivity.** A. The immune score differences of each immune microenvironment infiltrating immunocyte in groups of pSS patients with high and low anti-Ro/SSA antibody level in dataset #3. B. Correlation between YTHDC1 and immune score of immature B cells in pSS patients in dataset #3. C. The significantly enriched top 10 GO: BP terms on the differentially expressed genes between groups of pSS patients with high and low anti-Ro/SSA antibody level in dataset #3. D. The venn plot illustrates the intersection of genes mediated by the four m6A regulators (RBM15B, ZC3H13, YTHDC1 and YTHDF1) and the DEGs between groups of pSS patients with high and low anti-Ro/SSA antibody level. E. The significantly enriched top 10 GO: BP terms on the DEGs mediated by the four m6A regulators.


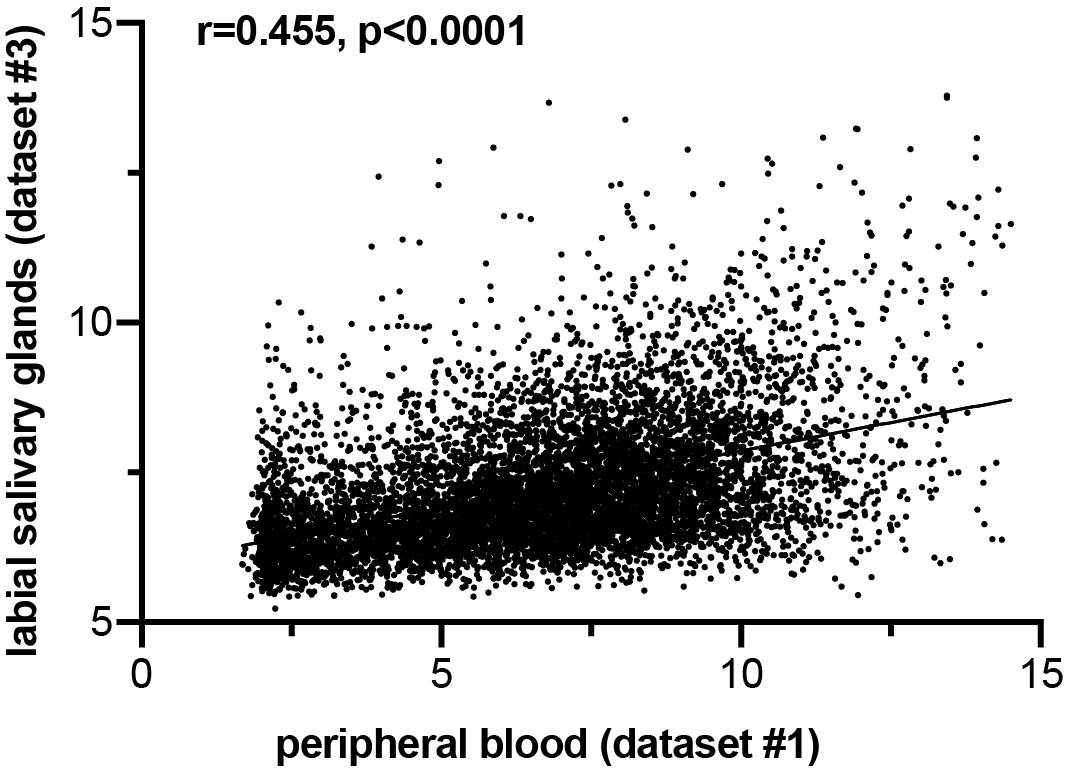


**Figure S7 Scatter plot demonstrating the correlation in gene expression between peripheral blood (dataset #1) and labial salivary glands (dataset #3).**
